# Hydrophobic Rose Bengal Derivatives Exhibit Submicromolar-to-Subnanomolar Activity against Enveloped Viruses

**DOI:** 10.3390/biom12111609

**Published:** 2022-11-01

**Authors:** Anna A. Rubekina, Polina N. Kamzeeva, Vera A. Alferova, Elena Yu. Shustova, Ekaterina S. Kolpakova, Elizaveta V. Yakovchuk, Evgenia V. Karpova, Maria O. Borodulina, Evgeny S. Belyaev, Alexei A. Khrulev, Vladimir A. Korshun, Evgeny A. Shirshin, Liubov I. Kozlovskaya, Andrey V. Aralov

**Affiliations:** 1 Faculty of Physics, M.V. Lomonosov Moscow State University, 1-2 Leninskie Gory, 119234 Moscow, Russia; 2 Shemyakin-Ovchinnikov Institute of Bioorganic Chemistry, Russian Academy of Sciences, Miklukho-Maklaya 16/10, 117997 Moscow, Russia; 3 Gause Institute of New Antibiotics, Russian Academy of Sciences, Bolshaya Pirogovskaya 11, 119021 Moscow, Russia; 4 Chumakov Scientific Center for Research and Development of Immune-and-Biological Products, Russian Academy of Sciences (Institute of Poliomyelitis), 108819 Moscow, Russia; 5 Frumkin Institute of Physical Chemistry and Electrochemistry, Russian Academy of Science, 119071 Moscow, Russia; 6 Laboratory of Clinical Biophotonics, Biomedical Science and Technology Park, I.M. Sechenov First Moscow State Medical University, Trubetskaya Str. 8-2, 119991 Moscow, Russia; 7 Institute of Translational Medicine and Biotechnology, Sechenov First Moscow State Medical University, Trubetskaya Str. 8-2, 119991 Moscow, Russia

**Keywords:** Rose Bengal, antiviral activity, photosensitization, ^1^O_2_ generation

## Abstract

Rose Bengal (RB) is an anionic xanthene dye with multiple useful biological features, including photosensitization properties. RB was studied extensively as a photosensitizer, mostly for antibacterial and antitumor photodynamic therapy (PDT). The application of RB to virus inactivation is rather understudied, and no RB derivatives have been developed as antivirals. In this work, we used a synthetic approach based on a successful design of photosensitizing antivirals to produce RB derivatives for virus photoinactivation. A series of n-alkyl-substituted RB derivatives was synthesized and evaluated as antiviral photosensitizers. The compounds exhibited similar ^1^O_2_ generation rate and efficiency, but drastically different activities against SARS-CoV-2, CHIKV, and HIV; with comparable cytotoxicity for different cell lines. Submicromolar-to-subnanomolar activities and high selectivity indices were detected for compounds with C_4-6_ alkyl (SARS-CoV-2) and C_6-8_ alkyl (CHIKV) chains. Spectrophotometric assessment demonstrates low aqueous solubility for C_8-10_ congeners and a significant aggregation tendency for the C_12_ derivative, possibly influencing its antiviral efficacy. Initial evaluation of the synthesized compounds makes them promising for further study as viral inactivators for vaccine preparations.

## 1. Introduction

Emerging and re-emerging viral pathogens are responsible for significant morbidity and pose a serious threat to public health around the world. Thus, the development of broad-spectrum antivirals that act simultaneously on a variety of structurally and phylogenetically diverse viruses is needed. However, currently approved broad-spectrum antivirals suffer from such disadvantages as poor bioavailability, low selectivity, and rapid body clearance [1,2,3,4]; thus, the research and development of new candidates is ongoing. In general, this type of anti-pathogenic agent targets a conserved pathway or proteins which different viruses use to infect host cells, or common structural features of the viruses. Among host targets, proteases [5], inosine monophosphate dehydrogenase (IMPDH) [6], and polymerases [7,8] are of noteThe outer lipid membrane, in turn, represents an attractive target shared by all enveloped viruses causing socially significant infections. Importantly, these membranes are not encoded by the viral genome; therefore, viral membrane-targeting antivirals would probably raise the barrier for variation-induced resistance.

Photosensitizing antivirals are a promising class of drug candidates against enveloped viruses [9]. Among a variety of structurally and spectrally distinct compounds [10], rigid amphipathic fusion inhibitors (RAFIs) [11,12,13,14,15] and thiazolidine derivatives [16,17] look especially attractive. These compounds selectively influence viral envelopes and inhibit viral fusion at nanomolar concentrations with no significant cytotoxicity, as the activity-determining mechanism of action for both classes is currently assumed to be the modification of phospholipids in the viral lipid bilayer via singlet oxygen (^1^O_2_) production under light exposure [17,18]. Recently, an effective ^1^O_2_-based virus inactivation strategy using thiazolidine derivatives for the preparation of inactivated vaccines has been proposed [19]. This approach avoids the disadvantages of conventional virus deactivators, such as modification of viral surface proteins and carcinogenesis for formalin [20,21,22] and toxicity for aziridine derivatives [23].

Rose Bengal (RB) is an anionic xanthene dye, a derivative of fluorescein, that demonstrated activity upon illumination against herpes simplex virus 1 [24,25], Friend leukemia virus [26], and Sendai, influenza, human immunodeficiency, and vesicular stomatitis viruses [27]. Medical applications of RB are mostly limited by its short half-life; therefore, various delivery systems have been developed [28]. RB found wide application in sono-photodynamic therapy acting predominantly via a type 2 photoreaction—generation of ^1^O_2_ [28]. Derivatization enables a wide array of possibilities for biological property tuning; nonetheless, for RB, derivatization efforts have mostly been limited to inactivation of bacteria [29,30] and cancer cells [31]. Here we report the antiviral activity, cell cytotoxicity, efficiency of ^1^O_2_ generation, and aqueous solubility of a series of RB derivatives with various hydrophobic n-alkyl chains.

## 2. Materials and Methods

### 2.1. General

All reagents and solvents were commercially available, unless otherwise mentioned, and used without further purification. ^1^H and ^13^C NMR spectra were registered on a Bruker Avance III 600 spectrometer at 600 and 150 MHz, respectively. Chemical shifts are given in δ (ppm) units relative to residual ^1^H or ^13^C signal from DMSO-*d*_6_ as reference. Multiplicity is shown using the following abbreviations: s for singlet, t for triplet, and m for multiplet. Coupling constants (J) are given in Hz. A Thermo Scientific LTQ Orbitrap hybrid instrument (Thermo Electron Corp., Bremen, Germany) was used to register ESI HR mass spectra in continuous flow direct sample infusion (positive ion mode).

### 2.2. Synthesis

To a solution of Rose Bengal, disodium salt **1** (1.0 g; 1.0 mmol) in DMF, the corresponding 1-bromoalkane (2.7 mmol) was added at room temperature in one portion. The resulting solution was stirred at 80 °C for 6 h. The reaction mixture was concentrated in vacuo, and the residue was stirred with diethyl ether (20 mL) overnight. The precipitate was filtered and the filter cake was washed with diethyl ether (2 × 5 mL), followed by the stirring with water (5 mL) overnight. The precipitate was filtered and dried in vacuo, affording ester **2** as a dark brown powder.

#### 2.2.1. Rose Bengal N-Butyl Ester, Monosodium Salt **2a**

Yield 91%. ^1^H NMR (600 MHz, DMSO-*d*_6_): δ 7.46 (s, 2H), 3.92 (t, *J* = 6.3 Hz, 2H), 1.12–1.06 (m, 2H), 0.95–0.88 (m, 2H), 0.64 (t, *J* = 7.4 Hz, 3H). ^13^C NMR (150 Hz, DMSO-*d*_6_): δ 171.7 (2C), 163.0, 156.9 (2C), 139.1, 135.9 (2C), 134.7, 134.2, 133.6, 131.7, 129.8, 128.6, 110.0 (2C), 97.1 (2C), 75.7 (2C), 65.8, 29.6, 18.3, 13.4. HRMS (ESI) *m*/*z*: calcd for C_24_H_13_Cl_4_I_4_O_5_^+^ [M-Na+2H]^+^: 1028.5690; found 1028.5675.

#### 2.2.2. Rose Bengal N-Hexyl Ester, Monosodium Salt **2b**

Yield 89%. ^1^H NMR (600 MHz, DMSO-*d*_6_): δ 7.47 (s, 2H), 3.92 (t, *J* = 6.4 Hz, 2H), 1.13–1.04 (m, 4H), 1.03–0.97 (m, 2H), 0.93–0.86 (s, 2H), 0.79 (t, *J* = 7.3 Hz, 3H). ^13^C NMR (150 Hz, DMSO-*d*_6_): δ 171.5 (2C), 163.0, 156.8 (2C), 139.1, 136.0 (2C), 134.7, 134.2, 133.6, 131.7, 129.7, 128.6, 110.4 (2C), 97.0 (2C), 75.8 (2C), 66.0, 30.6, 27.5, 24.5, 21.7, 13.9. HRMS (ESI) *m*/*z*: calcd for C_26_H_17_Cl_4_I_4_O_5_^+^ [M-Na+2H]^+^: 1056.6003; found 1056.5986.

#### 2.2.3. Rose Bengal N-Octyl Ester, Monosodium Salt **2c**

Yield 93%. ^1^H NMR (600 MHz, DMSO-*d*_6_): δ 7.50 (s, 2H), 3.92 (t, *J* = 6.3 Hz, 2H), 1.27–1.21 (m, 2H), 1.19–1.12 (m, 2H), 1.10–1.04 (m, 4H), 1.04–0.97 (m, 2H), 0.90–0.83 (m, 2H), 0.84 (t, *J* = 7.3 Hz, 3H). ^13^C NMR (150 Hz, DMSO-*d*_6_): δ 171.2 (2C), 163.0, 156.7 (2C), 139.2, 136.2 (2C), 134.8, 134.2, 133.6, 131.7, 129.6, 128.6, 110.8 (2C), 96.9 (2C), 75.9 (2C), 66.0, 31.1, 28.3, 28.2, 27.5, 24.8, 21.9, 13.9. HRMS (ESI) *m*/*z*: calcd for C_28_H_21_Cl_4_I_4_O_5_^+^ [M-Na+2H]^+^: 1084.6316; found 1084.6304.

#### 2.2.4. Rose Bengal N-Decyl Ester, Monosodium Salt **2d**

Yield 83%. ^1^H NMR (600 MHz, DMSO-*d*_6_): δ 7.45 (s, 2H), 3.92 (t, *J* = 6.4 Hz, 2H), 1.28–1.14 (m, 8H), 1.11–1.05 (m, 4H), 1.04–0.98 (m, 2H), 0.92–0.86 (m, 2H), 0.85 (t, – = 7.1 Hz, 3H). ^13^C NMR (150 Hz, DMSO-*d*_6_): δ 171.6 (2C), 163.0, 156.9 (2C), 139.1, 135.9 (2C), 134.7, 134.2, 133.6, 131.7, 129.8, 128.6, 110.1 (2C), 97.1 (2C), 75.8 (2C), 66.0, 31.2, 28.8, 28.6, 28.5, 28.4, 27.5, 24.8, 22.0, 13.8. HRMS (ESI) *m*/*z*: calcd for C_30_H_25_Cl_4_I_4_O_5_^+^ [M-Na+2H]^+^: 1112.6629; found 1112.6621.

#### 2.2.5. Rose Bengal N-Dodecyl Ester, Monosodium Salt **2e**

Yield 72%. ^1^H NMR (600 MHz, DMSO-*d*_6_): δ 7.45 (s, 2H), 3.91 (t, *J* = 6.4 Hz, 2H), 1.28–1.14 (m, 12H), 1.11–1.04 (m, 4H), 1.04–0.98 (m, 2H), 0.91–0.85 (m, 2H), 0.85 (t, *J* = 7.1 Hz, 3H). ^13^C NMR (150 Hz, DMSO-*d*_6_): δ 171.6 (2C), 163.0, 156.9 (2C), 139.1, 136.0 (2C), 134.7, 134.2, 133.6, 131.7, 129.8, 128.6, 110.1 (2C), 97.1 (2C), 75.8 (2C), 66.0, 31.2, 28.9, 28.9, 28.8, 28.6, 28.5, 28.4, 27.5, 24.8, 22.0, 13.8. HRMS (ESI) *m*/*z*: calcd for C_32_H_29_Cl_4_I_4_O_5_^+^ [M-Na+2H]^+^: 1140.6942; found 1140.6929.

### 2.3. Assessment of ^1^O_2_ Generation

Absorption spectra were measured in the 300–750 nm spectral range using a UV–Vis Lambda 25 spectrophotometer (Perkin Elmer, Waltham, MA, USA). Steady-state fluorescence measurements were performed on a FluoroMax-4 spectrofluorometer (HORIBA Jobin Yvon SAS, Longjumeau, France). Fluorescence spectra in the 515–650 nm range were measured at the 504 nm excitation wavelength. The spectral width of excitation and emission slits was set to 1 nm.

1 μM solutions of RB **1** or its derivatives **2a**–**e** in methanol were used to test the photosensitizing ability. To determine the efficiency of ^1^O_2_ generation, commercially available fluorescent dye SOSG (Singlet Oxygen Sensor Green, Thermo-Fisher Scientific, Madison, WI, USA) at the final concentration of 10 μM was used (https://tools.thermofisher.com/content/sfs/manuals/mp36002.pdf, accessed on 17 October 2022). All samples were photo-oxidized by irradiation with LED (λ = 520 nm, intensity 10 mW/cm^2^) for 10 min at room temperature. Fluorescence spectra were measured every 30 s during irradiation. Solutions of (±)-α-tocopherol (Sigma-Aldrich, St. Louis, MO, USA) in methanol at 0.1 or 1 mg/mL concentrations were used to study its ability to quench ^1^O_2_ generation caused by RB **1**.

### 2.4. Solubility

Weighted samples were dissolved in DMSO to a 5 mM concentration and then diluted with phosphate-buffered saline (PBS) to a 50 μM concentration (PBS containing 1% DMSO). For RB **1**, an additional 10-fold dilution in PBS containing 1% DMSO was performed to a 5 μM concentration. The suspensions were sonicated for 5 min, shaken for 30 min, and centrifuged at 12,000 rpm for 10 min. The supernatants were photometrically analyzed and concentrations of **1**, **2a**, and **2b** were roughly evaluated, taking the molar absorption coefficient to be equal to 95,000 [32]. Since derivatives **2c**–**e** seem to form aggregates, causing two absorption maxima to appear, their concentration has not been evaluated.

### 2.5. Biological Activity

#### 2.5.1. Cells and Viruses

Vero cells originate from WHO Biologicals (Geneva, Switzerland). RD cells originate from the NIBSC (UK) cell collection (Accession No. 081003). MT-4 cells were obtained via the NIH AIDS Research and Reference Reagent Program (ARP-120).

SARS-CoV-2 strain PIK35 (GISAID EPI_ISL_428852), CHIKV strain Nic (GenBank IDs MN271691-2), Enterovirus A Coxsackievirus A16 (CVA16) isolate 49360 (GenBank ID MK704491), Enterovirus B Echovirus 13 (E13) isolate 57088 (GenBank ID MK704490), and Enterovirus C vaccine strain Sabin 1 of poliovirus type 1 (PV1, GenBank ID AY184219) were stored as infected cell suspension at −70 °C and used for cytopathic effect inhibition tests. HIV-1 strain NL4-3 was obtained via cell transfection with pNL4-3 (ARP2006, NIBSC, Potters Bar, UK).

#### 2.5.2. Cell Viability Assay (for Vero Cells)

Eight two-fold dilutions of RB **1** or its derivatives **2a**–**e** (5 mM or 0.2 mM stock solutions), and DMSO as a negative control, were prepared in DMEM (Chumakov FSC R&D IBP RAS, Russia). Compound dilutions were added to confluent Vero cell monolayers in 2 replicates. The final concentration series of eight dilutions started from 100 µM. After incubation at 37 °C in a CO_2_-incubator for 5 days, the culture medium was substituted with a resazurin solution (0.15 mg/mL). Cells were incubated at 37 °C in a CO_2_-incubator for 4 h. Then, 20 µL of 10% SDS were added to stop the reaction. Fluorescence was measured with Fluoroskan (ThermoFisher Scientific) at λ_ex_ = 544 nm and λ_em_ = 590 nm. Statistical analysis and fluorescence curves were prepared with Microsoft Excel 2013. The 50% cytotoxic concentration (CC_50_) was calculated (compound concentration required to induce reduction of fluorescence by 50%).

#### 2.5.3. Cell Viability Assay (for RD and MT-4 Cells)

Two-fold dilutions of RB **1** or its derivatives **2a**–**e** (5 mM stock solutions), and DMSO as a negative control, were prepared in an appropriate cell culture medium (Chumakov FSC R&D IBP RAS, Russia). MT-4 or RD cell suspensions were added to the wells with compound dilutions and the DMSO control (approximately 2 × 10^4^–1 × 10^5^ cells per well). After incubation at 37 °C in a CO_2_-incubator for 7 or 5 days, respectively, cells were analyzed by microscopy. Cytotoxic concentration (CC_50_) values were calculated using the Kärber method [21].

#### 2.5.4. Virus-Induced Cytopathic Effect Inhibition Test (SARS-CoV-2, CHIKV)

The procedure is as previously described [33]. In brief, eight 2-fold dilutions of 5 mM (or 100 µM) DMSO stock solutions of RB **1** or its derivatives **2a**–**e** were prepared in DMEM (Chumakov FSC R&D IBP RAS, Russia). Compound dilutions were mixed with equal volumes of a virus suspension containing 100 50% cell culture infectious doses (CCID_50_) per well. After 1 h incubation at 37 °C, virus-compound mixtures were added to confluent Vero cell monolayers in 2 replicates. Control cells were treated with the same sequential concentrations of DMSO as in the compound dilutions (negative control), or with N^4^-hydroxycytidine (NHC) [34] (positive control). After a 5-day incubation at 37 °C, the cytopathic effect (CPE) was visually assessed via microscope. EC_50_ values were calculated using the Kärber method [35]. The experiment was repeated at least 2 times for each compound. Each experiment included virus dose titration to assure an acceptable dose range.

#### 2.5.5. Cytopathic Effect Inhibition Test (HIV)

The procedure is as previously described [36], with some changes. In brief, eight two-fold dilutions of 5 mM stock solutions of the compounds in RPMI-1640 (Chumakov FSC R&D IBP RAS, Russia) were prepared in two replicates. Compound dilutions were mixed with equal volumes of a virus suspension containing 100 CCID_50_. Control cells were treated with the same sequential concentrations of DMSO as in the compound dilutions (negative control), or with AZT (positive control). Then, an MT-4 cell suspension (approx. 2 × 10^4^ cells per well) in RPMI-1640 containing 10% FBS (Invitrogen, South America) was added to the experimental mixtures. The final concentration series started from 50 µM. Each experiment included titration of the virus dose in the inoculate to assure an acceptable dose range. After a 7-day incubation (5% CO_2_, 37 °C), CPE was visually assessed via microscope. EC_50_ values were calculated using the Kärber method [21]. All experimental procedures were performed in two replicates and were repeated twice.

#### 2.5.6. Cytopathic Effect Inhibition Test (CVA16, E13 and PV1)

The procedure is as previously described [37]. In brief, eight 2-fold dilutions of 5 mM stock solutions of the compounds in 2 × EMEM (Chumakov FSC R&D IBP RAS, Russia) were prepared in two replicates to obtain the final concentration series starting from 100 μM. Compound dilutions were mixed with equal volumes of an enterovirus suspension containing 100 CCID_50_. Control cells were treated with the same sequential concentrations of DMSO as in the compound dilutions (negative control), or with N^4^-hydroxycytidine (NHC) [22] (positive control). After 1 h incubation at 36.5 °C, an RD cell suspension (approx. 1 × 10^5^ cells per well) in 2 × EMEM containing 5% FBS was added to the compound-virus mixtures. Each experiment included titration of the virus dose in the inoculate to assure an acceptable dose range. After a 5-day incubation at 37 °C, CPE signs were visually assessed via microscope. EC_50_ values were calculated using the Kärber method [21].

## 3. Results and Discussion

**Scheme 1:**
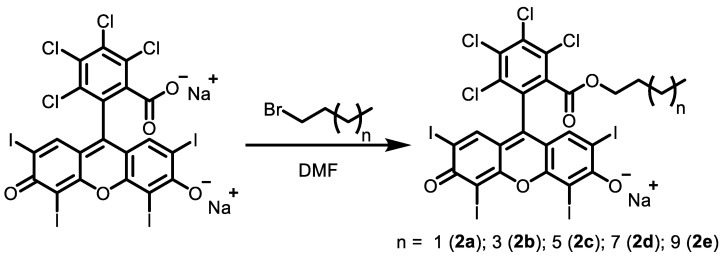
Preparation of n-alkyl-substituted RB derivatives.

Here, we synthesized five derivatives **2a**–**e** of a known photosensitizer, Rose Bengal (RB) **1** [38], containing an alkyl substituent of various lengths (from C4 to C12) at the RB carboxyl moiety according to a reported method [39,40,41]. Briefly, a mixture of RB **1** and the corresponding 1-bromoalkane was heated at 80 °C for 6 h and concentrated, and the starting materials were removed by sequential stirring in diethyl ether and water, followed by filtration and drying in vacuo, affording target derivatives **2a**–**e** (Scheme 1). The structure and the elemental composition of **2a**–**e** were confirmed by NMR spectroscopy and HRMS spectrometry, respectively (see Section 2.2 *Synthesis* and Figure S3).

Antiviral activity of the synthesized derivatives **2a**–**e** against SARS-CoV-2/CHIKV was evaluated on Vero cells, while MT-4 and RD cells were used for HIV and enteroviruses (CVA16/E13/PV1) (Table S1), respectively. RB **1** and N^4^-hydroxycytidine (NHC) [34] or azidothymidine (AZT) [42,43] were used as positive controls with the same (RB) or different (lethal mutagenesis for NHC [44] and reverse transcriptase inhibition for AZT [42]) mechanisms of action. Among the compounds studied, **2a** exhibited activity against SARS-CoV-2 at nanomolar concentrations and was superior to RB **1** in terms of activity and SI, while **2b** has activity comparable to RB, but with a higher SI (Table 1), suggesting the optimal alkyl substituent length of C4 (n-butyl) for this virus. As the length of the alkyl substituent in the series increases further, a reduction in activity and SI is observed. For CHIKV, the most potent derivatives **2b** and **2c,** with n-hexyl (C6) and n-octyl (C8) tethers, respectively, demonstrated activity in subnanomolar concentrations and a two orders of magnitude higher efficiency compared to RB **1**. Despite the presence of some cytotoxicity in the micromolar range for Vero cells, low effective concentrations provide significant SI (for example, about 230,000 for **2c**), making these RB derivatives promising as deactivators in the development of vaccines against the Chikungunya virus [45]. All tested compounds also show activity against HIV; however, it is comparable to the observed cytotoxicity for MT-4 cells. RB has much lower activity against HIV than reported previously [27], which seems to be caused by the lack of preliminary illumination before the treatment of the cells. Finally, the compounds failed to inhibit nonenveloped enteroviruses like CVA16 [46], E13 and PV1 [47] (Table S1), thus supporting the viral membrane-dependent mechanism of action common to this class of antivirals [17].

Several factors may contribute to observed activity against the studied viruses and cell cytotoxicity, including the ability of the derivatives to generate ^1^O_2_, their solubility in physiological media and formation of dimers or aggregates.

**Figure 1:**
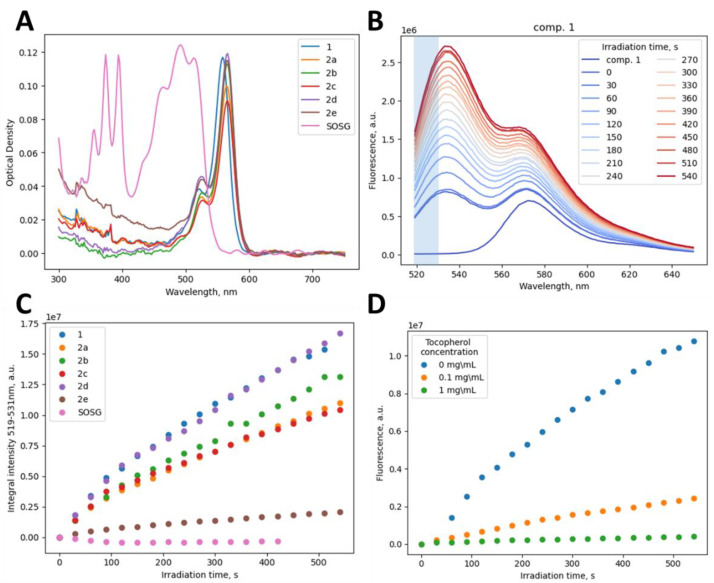
Steady-state optical properties of the investigated compounds: (**A**) Absorption spectra of non-irradiated samples; (**B**) typical fluorescence spectra of an irradiated sample containing RB **1** and SOSG (the integration area (519–530 nm) is highlighted with blue); (**C**) integral fluorescence intensity in the 519–530 nm spectral range as a function of photo-oxidation time; (**D**) integral fluorescence intensity in the 519–530 nm spectral range as a function of photo-oxidation time of SOSG and **2a** in methanol with or without (±)-α-tocopherol.

First, the ability of RB **1** and its derivatives **2a**–**e** to generate ^1^O_2_ was evaluated (Figure 1). The absorption spectra of the compounds and SOSG in methanol in the visible spectral range are shown in Figure 1A. The optical density of all samples was close to 0.12, they had the same spectral shape with the maximum at approximately 565 nm. They had non-zero optical density at 520 nm; therefore, their irradiation at 520 nm should lead to ^1^O_2_ generation. SOSG can also be a photosensitizer, so we first checked whether the dye could generate a significant amount of ^1^O_2_ under experimental conditions. The fluorescence intensity of SOSG did not change during irradiation, suggesting the increase in the dye’s fluorescence was associated solely with ^1^O_2_ generation by RB **1** and its derivatives (Figure S1).

Samples were irradiated at 520 nm for the specified lengths of time, and then fluorescence spectra were recorded at the 504 nm excitation wavelength (Figure 1B). The amount of generated ^1^O_2_ can be estimated from integral intensity in the 519–531 nm range, where only the signal from SOSG is detected, and the intrinsic fluorescence of RB and its derivatives is close to 0.

Integral fluorescence as a function of ^1^O_2_ generation time is shown in Figure 1C. By approximating the data with an exponential function (Equation (1)), it is possible to compare RB and its derivatives in terms of ^1^O_2_ generation rate (k) and the amount of ^1^O_2_ generated (amplitude, A) (Table 2). The ^1^O_2_ generation rate is approximately the same for all compounds, but the amount of generated ^1^O_2_ (directly proportional to the amplitude A) is slightly different for all compounds except **2e**. For derivative **2e**, it was minimal, while the concentration (C_gen_) was the highest, suggesting that **2e** is the least effective photosensitizer (Table 2). These results are consistent with **2e** having the lowest antiviral activity in the series (Table 1). Taking into account the equal ability to generate ^1^O_2_, the hydrophobicity of the tether might be the main parameter influencing the antiviral activity against SARS-CoV-2 and CHIKV. For HIV and cells, alkyl length has practically no effect on activity and cytotoxicity, respectively (Table 1).

(1) $$I_{519-530\;\mathrm{nm}}=C-A\times e^{-kt}$$

The effect of (±)-α-tocopherol, a well-known antioxidant [48], on the photosensitization ability of **2a**, the derivative with the optimum combined activity against SARS-CoV-2 and CHIKV, was also studied. ^1^O_2_ generation was determined the same way as in previous experiments. The results are shown in Figure 1D. The presence of (±)-α-tocopherol reduced the efficiency of ^1^O_2_ generation, reaching a 2 orders of magnitude reduction for the tocopherol concentration of 1 mg/mL. Thus, the adverse effects of RB derivatives on host cells can likely be mitigated by the presence of antioxidants within organisms [49].

The next factor that could affect the observed activity of the compounds is their solubility in aqueous media used to assess antiviral activity and cytotoxicity. Solubility in PBS containing 1% DMSO was evaluated spectrophotometrically (Figure S2). Similarly to RB **1**, derivatives **2a** and **2b** exist in a mixture of monomeric (λ_max_ 549 nm) and dimeric (λ_max_ 515 nm) forms [50], with the proportion of dimers increasing with higher alkyl chain lengths. For this reason, we made a rough estimate the solubility based on absorption intensity for the monomeric form at 549 nm, judging that the dimeric form is not a photosensitizer [50] and likely does not contribute to ^1^O_2_-mediated antiviral activity. The saturation concentration (C_sat_) for RB **1** and **2a**–**b** was significantly higher than EC_50_ for SARS-CoV-2 and CHIKV and comparable to EC_50_ for HIV (Table 1). Derivatives **2c**–**e** seem to form higher aggregates, which lead to the lowest excited singlet state energy level being split and two absorption maxima appearing at higher (λ_max_ 568 nm) and lower (λ_max_ 532 nm) wavelengths (549 nm) [51]. The formation of dimers/aggregates does not allow estimating the solubility of **2c**–**e**, but the area under curve (AUC) for **2e** is higher than for **2c**–**d** and even **2b**, which did not form any supramolecular assemblies. Thus, although **2e** appears to have higher solubility than derivatives **2b**–**d** with shorter alkyl substituents, its amount of generated ^1^O_2_ being the lowest in the series is responsible for its lower antiviral activity and cell cytotoxicity (Table 1 and Table 2). To conclude this part, while RB **1** and its congeners **2a**–**b** with shorter (C4 and C6) alkyl substituents have C_sat_ higher than or comparable with EC_50_, low solubility and aggregate formation have likely affected the observed antiviral activity of **2d**–**e**, with longer (C10 and C12) alkyl groups.

To conclude, the effect of the introduction of fatty alkyl (C4–C12) substituents at the carboxy functionality of Rose Bengal on cell cytotoxicity and activity against a number of enveloped viruses was studied. Photophysical studies and the lack of activity against non-enveloped viruses indirectly support the mechanism of action associated with the impact of generated ^1^O_2_ on viral membranes. A reduction in aqueous solubility and aggregate formation with increasing alkyl group length may affect antiviral activity. The observed submicromolar-to-subnanomolar activity against SARS-CoV-2 and CHIKV and the extremely high selectivity index for alkyl-modified RB derivatives opens up an opportunity to develop broad-spectrum viral inactivants for vaccine preparations.

## Figures and Tables

**Table 1:** Antiviral and cytotoxic properties of the studied compounds.

Cmpd	EC_50_, µM (M ± SD) ^a^			CC_50_, µM (M ± SD) ^b^		SI ^c^		
	SARS-CoV-2	CHIKV	HIV	Vero Cells	MT-4 Cells	SARS-CoV-2	CHIKV	HIV
**1**	0.5 ± 0.2	0.11 ± 0.05	18 ± 7	43 ± 10	>50	91	391	>3
**2a**	0.05 ± 0.02	0.004 ± 0.002	6.3 ± 2.6	8 ± 1	15 ± 4	169	1860	2.4
**2b**	0.4 ± 0.3	0.0007 ± 0.0003	4.4 ± 1.8	60 ± 15	15 ± 4	146	90,909	3.4
**2c**	5.3 ± 1.3	0.0002 ± 0.0001	8.8 ± 3.7	53 ± 25	42 ± 10	10	230,435	4.8
**2d**	6.6 ± 3.1	0.002 ± 0.001	8.8 ± 2.6	60 ± 15	42 ± 10	9	28,571	4.8
**2e**	43 ± 10	0.038 ± 0.030	>50	>100	>50	>2	>2632	n/d
Positive control	4.7 ± 2.9 ^d^	23.6 ± 10.2 ^d^	0.01 ^e^	>100	>5	>21	>4	>455

^a^ Effective concentration required to inhibit virus-induced cytopathic effect by 50%. ^b^ Compound concentration required to induce a reduction of fluorescence by 50%. ^c^ Selectivity index (SI) is the ratio of CC_50_ for the appropriate cell line to EC_50_ for the studied virus. ^d^ NHC was used as a positive control. ^e^ AZT was used as a positive control.

**Table 2:** Photophysical properties and solubility of the studied compounds.

Cmpd	^1^O_2_ Generation			Solubility
	C_gen_, μM ^a^	k, ×10^−3^ 1/s ^b^	A, ×10^−7^ a.u.f ^c^	C_sat_, μM ^d^
**1**	1.3	1.86	3.23	>50.0
**2a**	1.2	1.43	2.28	3.1
**2b**	1.3	0.97	4.04	1.0
**2c**	1.5	2.23	1.98	ND
**2d**	1.1	1.29	3.54	ND
**2e**	2.3	2.21	0.635	ND

^a^ Compound concentration used in ^1^O_2_ generation experiments. ^b 1^O_2_ generation rate. ^c^ Amplitude is a measure of ^1^O_2_ generation efficiency proportional to the amount of ^1^O_2_ bound by SOSG. ^d^ Saturation concentration of RB and its derivatives in PBS containing 1% DMSO.

## Data Availability

Not applicable.

## Supplementary Materials

The following supporting information can be downloaded at: https://www.mdpi.com/article/10.3390/biom12111609/s1, Table S1: Antiviral activity of RB and 2a–e against non-enveloped viruses and cytotoxicity for RD cells; Figure S1: Fluorescence spectra of SOSG during LED irradiation (520 nm); Figure S2: Absorption spectra of RB 1 (an additional 10-fold dilution) and its derivatives 2a–e in PBS containing 1% DMSO, used for solubility evaluation; Figure S3: NMR spectra.
