# Supplementary material for: Hydrophobic Rose Bengal Derivatives Exhibit Submicromolar-to-Subnanomolar Activity against Enveloped Viruses
Source: Biomolecules. 2022 Nov 1;12(11):1609. doi: 10.3390/biom12111609 (PMC9687286; doi:10.3390/biom12111609)
Supplement: Supplementary file 1 [file biomolecules-12-01609-s001.zip › biomolecules-2010459-supplementary.pdf]

# Hydrophobic Rose Bengal derivatives exhibit submicromolar-to-subnanomolar activity against enveloped viruses

Anna A. Rubekina,[1,†] Polina N. Kamzeeva,[2,†] Vera A. Alferova,[2,3] Elena Yu. Shustova,[4] Ekaterina S. Kolpakova,[4] Elizaveta V. Yakovchuk,[4] Evgenia V. Karpova,[4] Maria O. Borodulina,[4] Evgeny S. Belyaev,[5] Alexei A. Khrulev,[2] Vladimir A. Korshun,[2] Evgeny A. Shirshin,[1,6] Liubov I. Kozlovskaya,[4,7] and Andrey V. Aralov[2,*]

# Supplementary information

[1] Faculty of Physics, M.V. Lomonosov Moscow State University, 1-2 Leninskie Gory, 119234 Moscow, Russia

[2] Shemyakin-Ovchinnikov Institute of Bioorganic Chemistry, Miklukho-Maklaya 16/10, Moscow 117997, Russia

[3] Gause Institute of New Antibiotics, B. Pirogovskaya 11, 119021 Moscow, Russia,

[4] Chumakov Scientific Center for Research and Development of Immune-and-Biological Products, Russian Academy of Sciences (Institute of Poliomyelitis), Moscow, 108819, Russia

[5] Frumkin Institute of Physical Chemistry and Electrochemistry of the Russian Academy of Science, Moscow, 119071, Russia

[6] Laboratory of Clinical Biophotonics, Biomedical Science and Technology Park, I.M. Sechenov First Moscow State Medical University, Trubetskaya Str. 8-2, Moscow 119991, Russia

[7] Sechenov First Moscow State Medical University, Trubetskaya Str. 8-2, 119991 Moscow, Russia

* Correspondence: Baruh238@mail.ru

[†] These authors contributed equally to this work

## Contents

Table S1. Antiviral activity of RB and 2a-e against non-enveloped viruses and cytotoxicity for RD cells ........ 3

Figure S1. Fluorescence spectra of SOSG during LED irradiation (520 nm) ........ 4

Figure S2. Absorption spectra of RB 1 (an additional 10-fold dilution) and its derivatives 2a-e in PBS containing 1% DMSO, used for solubility evaluation ........ 5

Figure S3. NMR spectra. ........ 6

Rose Bengal n-Butyl Ester, Monosodium Salt **2a,** $^{1}$H spectrum ........ 6

Rose Bengal n-Butyl Ester, Monosodium Salt **2a,** $^{13}$C spectrum ........ 6

Rose Bengal n-Hexyl Ester, Monosodium Salt **2b,** $^{1}$H spectrum ........ 7

Rose Bengal n- Hexyl Ester, Monosodium Salt **2b,** $^{13}$C spectrum ........ 7

Rose Bengal n- Octyl Ester, Monosodium Salt **2c,** $^{1}$H spectrum ........ 8

Rose Bengal n- Hexyl Ester, Monosodium Salt **2b,** $^{13}$C spectrum ........ 8

Rose Bengal n-Decyl Ester, Monosodium Salt **2d,** $^{1}$H spectrum ........ 9

Rose Bengal n-Decyl Ester, Monosodium Salt **2d,** $^{13}$C spectrum ........ 9

Rose Bengal n-Dodecyl Ester, Monosodium Salt **2e,** $^{1}$H spectrum ........ 10

Rose Bengal n-Dodecyl Ester, Monosodium Salt **2e,** $^{13}$C spectrum ........ 10

**Table S1. Antiviral activity of RB and 2a-e against non-enveloped viruses and cytotoxicity for RD cells**

| Cmpd | $EC_{50}$, µM (M ± SD)[a] | | | $CC_{50}$, µM (M ± SD)[b] |
|---|---|---|---|---|
| | **CVA16** | **E13** | **PV1** | **RD cells** |
| **1** | >100 | >100 | >100 | 43 ± 10 |
| **2a** | >100 | >100 | >100 | 21.3 ± 5.2 |
| **2b** | >100 | >100 | >100 | 21.3 ± 5.2 |
| **2c** | >100 | >100 | >100 | 21.3 ± 5.2 |
| **2d** | >100 | >100 | >100 | 10.7 ± 2.6 |
| **2e** | >100 | >100 | >100 | 21.3 ± 5.2 |
| NHC | 50 ± 18 | 17 ± 8 | 25 ± 10 | >100 |

[a] Effective concentration required to inhibit virus-induced cytopathic effect by 50%

[b] Compound concentration required to induce a reduction of fluorescence by 50%

Back to Contents

**Figure S1. Fluorescence spectra of SOSG during LED irradiation (520 nm)**

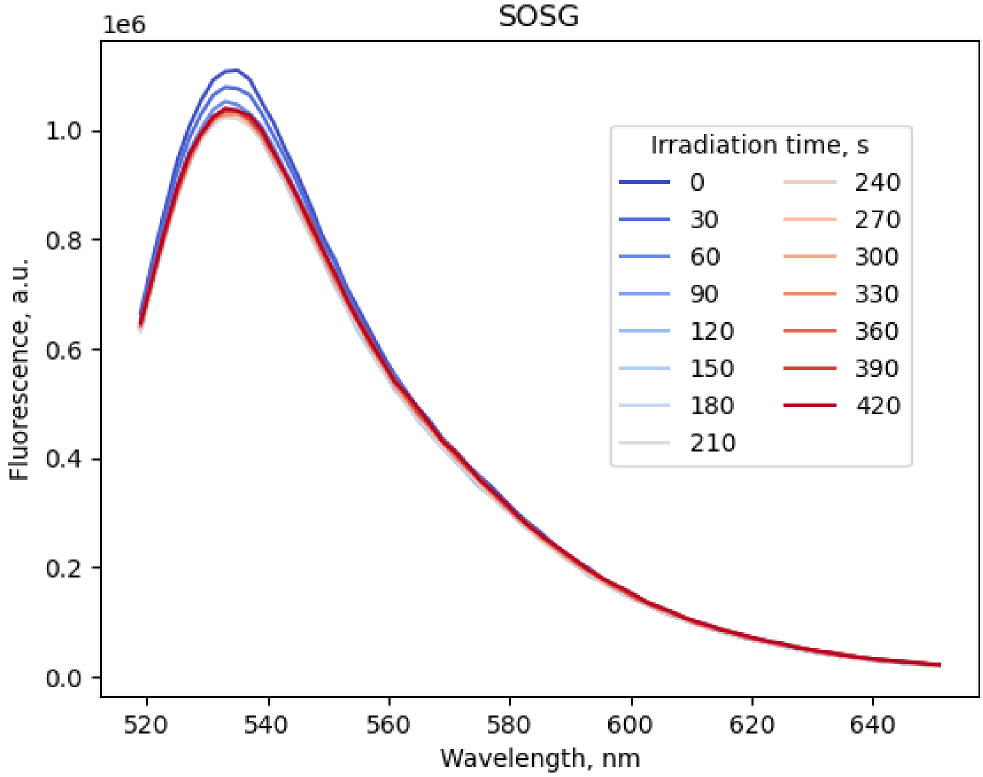


Back to Contents

**Figure S2. Absorption spectra of RB 1 (an additional 10-fold dilution) and its derivatives 2a-e in PBS containing 1% DMSO, used for solubility evaluation**

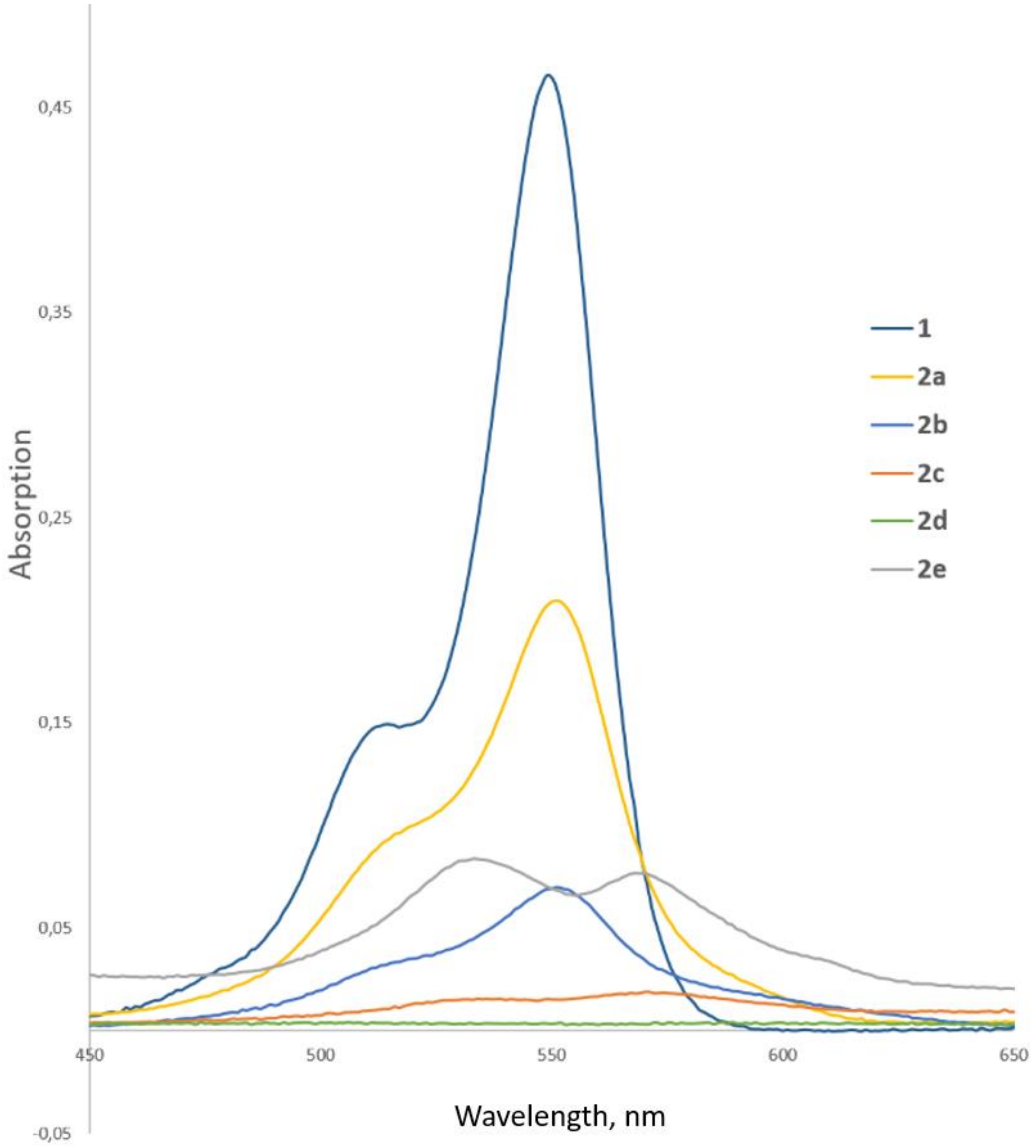


Back to Contents

**Figure S3. NMR spectra.**

Rose Bengal n-Butyl Ester, Monosodium Salt **2a,** $^{1}$H spectrum

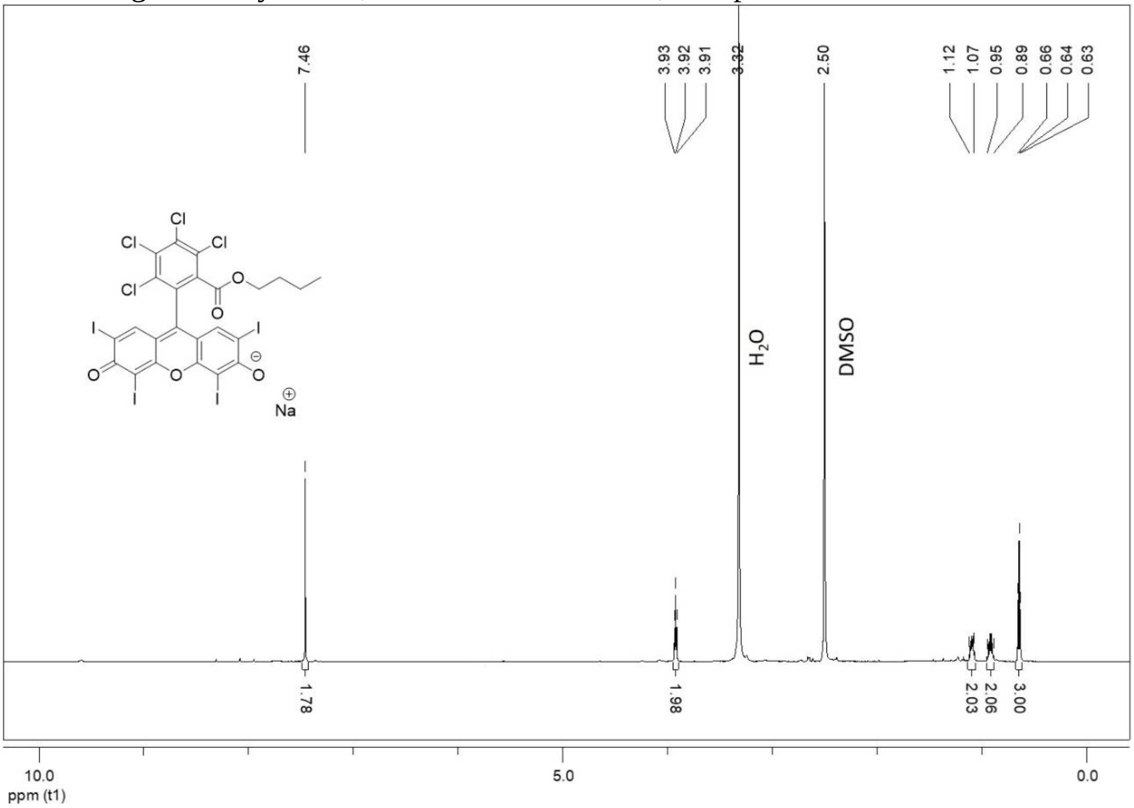


Rose Bengal n-Butyl Ester, Monosodium Salt **2a,** $^{13}$C spectrum

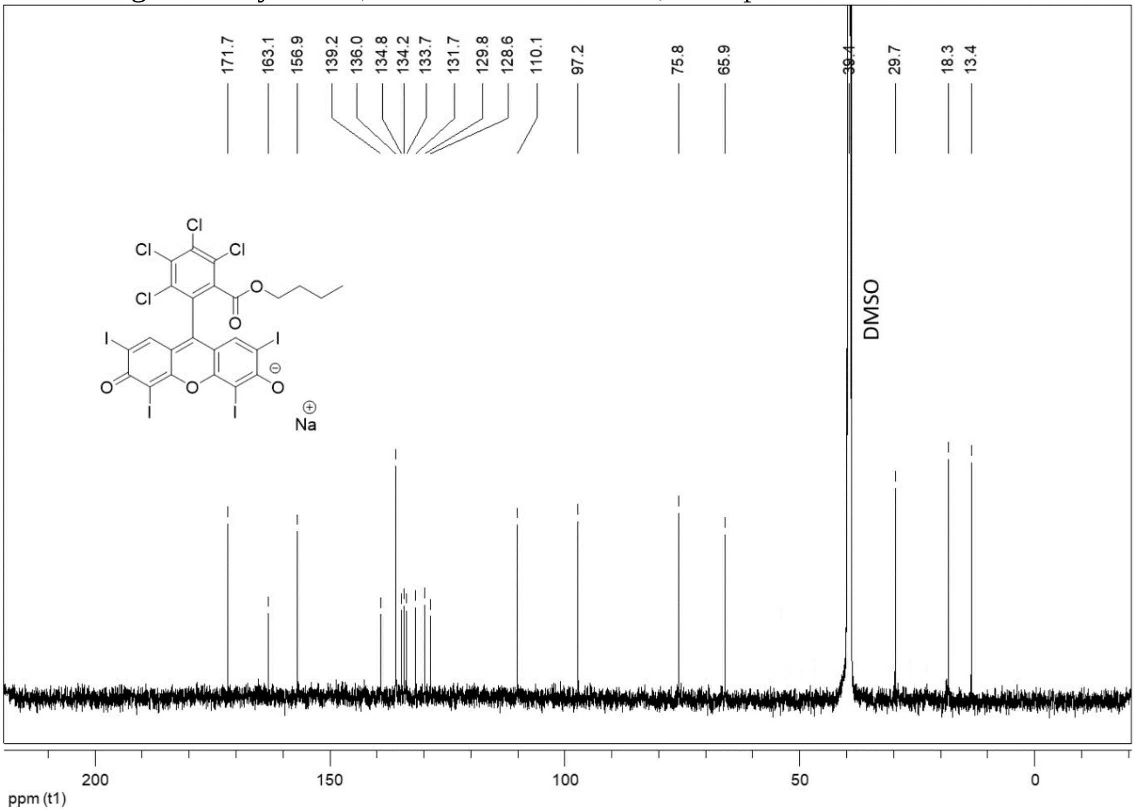


[Back to Contents]

Rose Bengal n-Hexyl Ester, Monosodium Salt **2b,** $^{1}$H spectrum

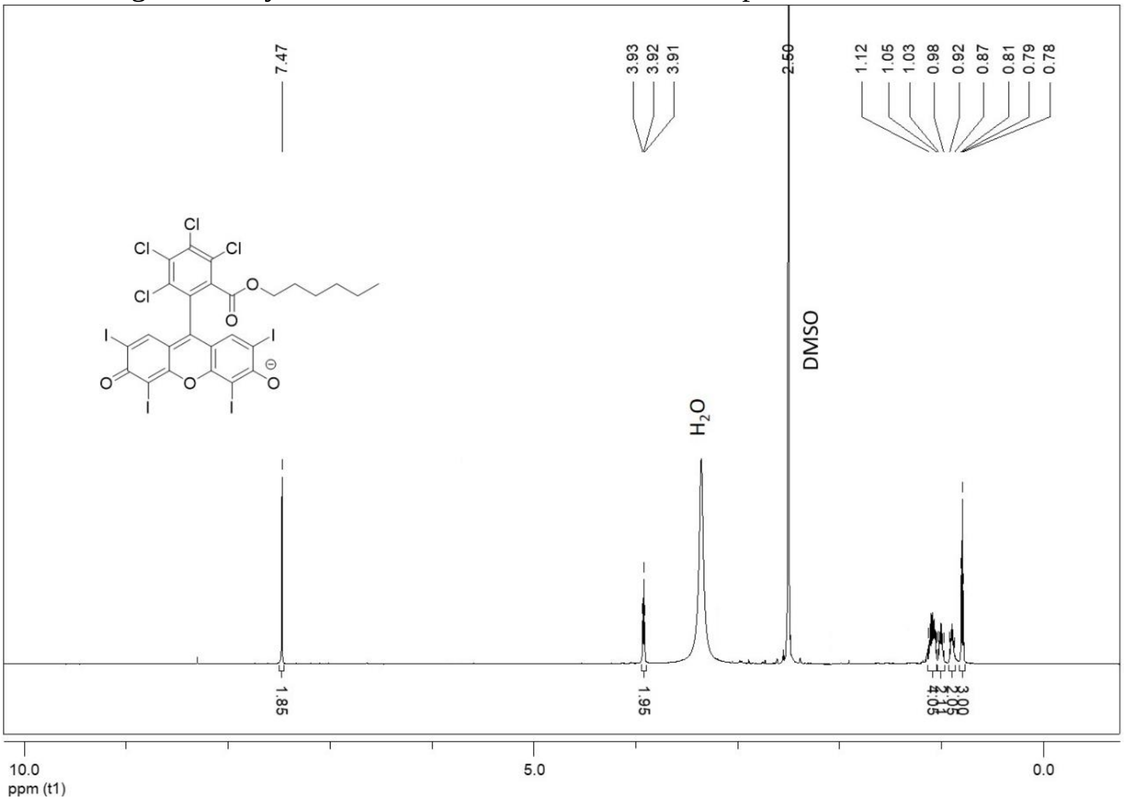


Rose Bengal n- Hexyl Ester, Monosodium Salt **2b,** $^{13}$C spectrum

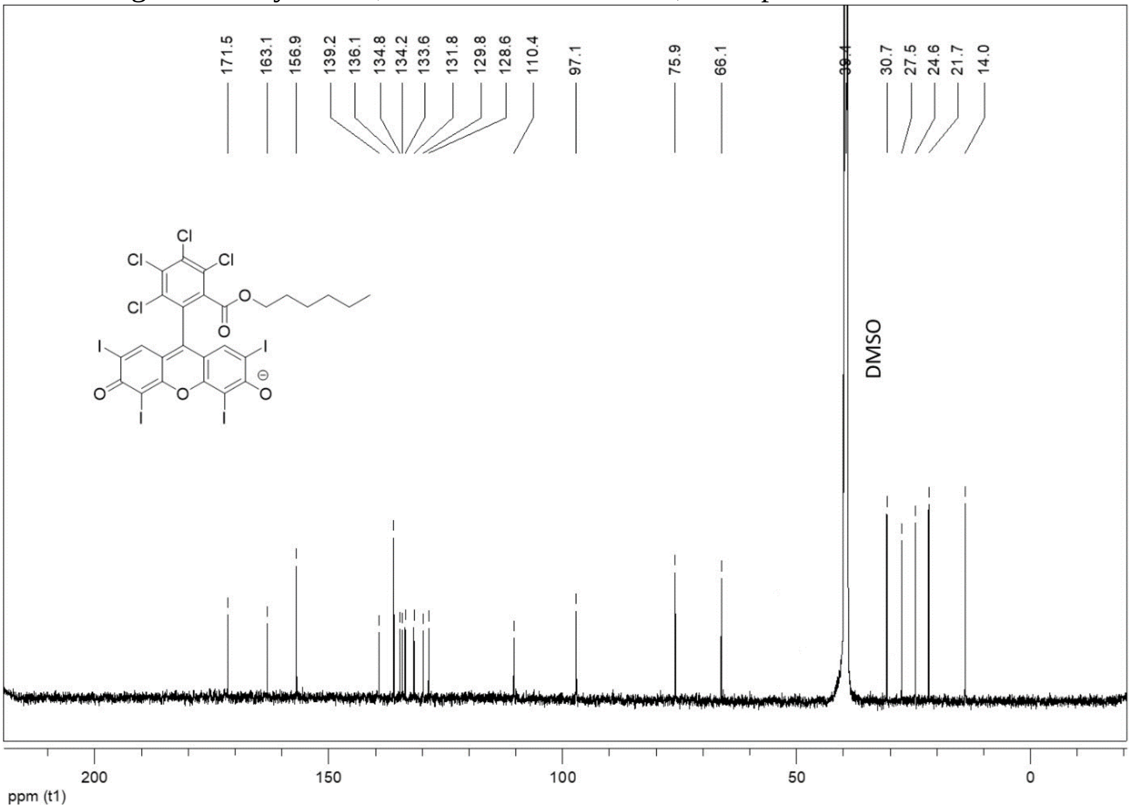


Back to Contents

Rose Bengal n- Octyl Ester, Monosodium Salt **2c,** $^{1}H$ spectrum

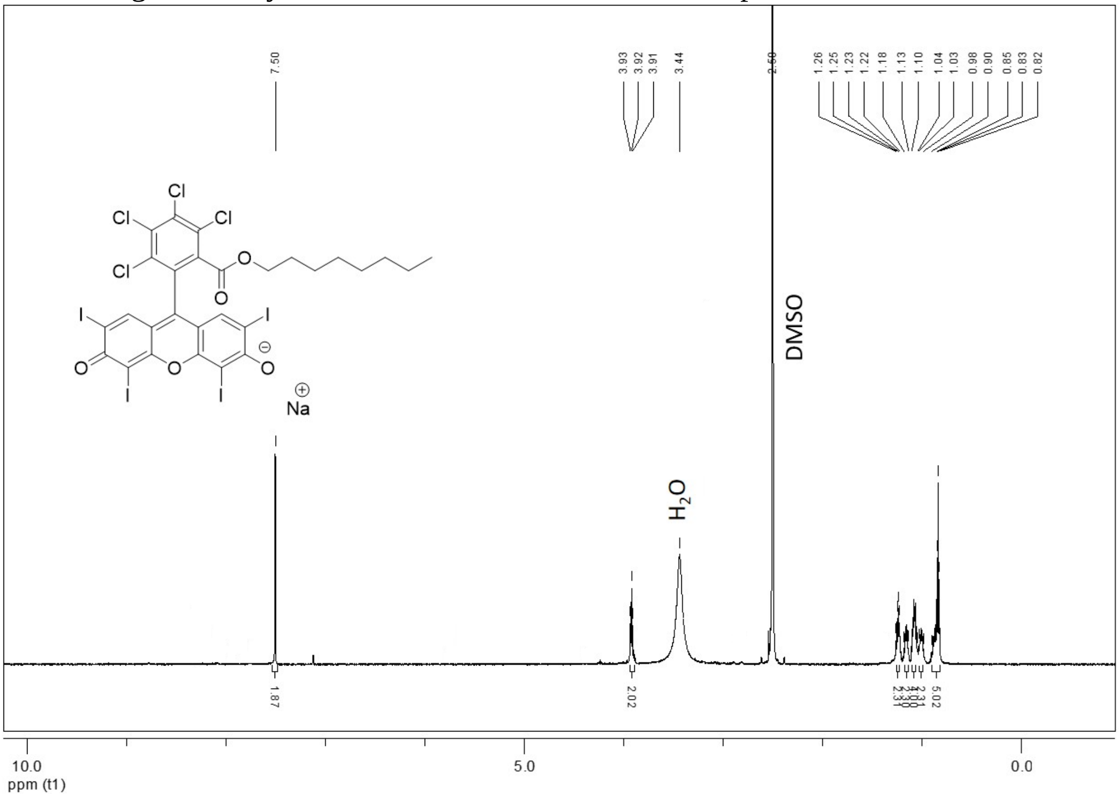


Rose Bengal n- Hexyl Ester, Monosodium Salt **2b,** $^{13}C$ spectrum

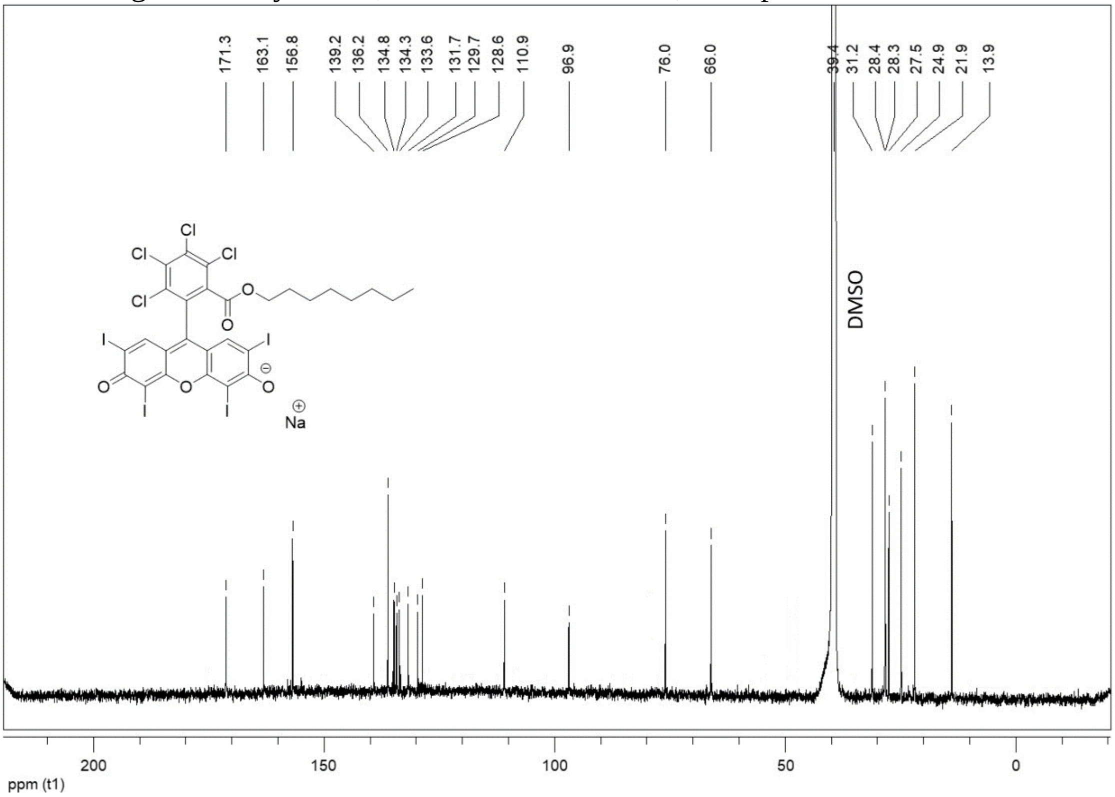


Back to Contents

Rose Bengal n-Decyl Ester, Monosodium Salt **2d,** $^{1}$H spectrum

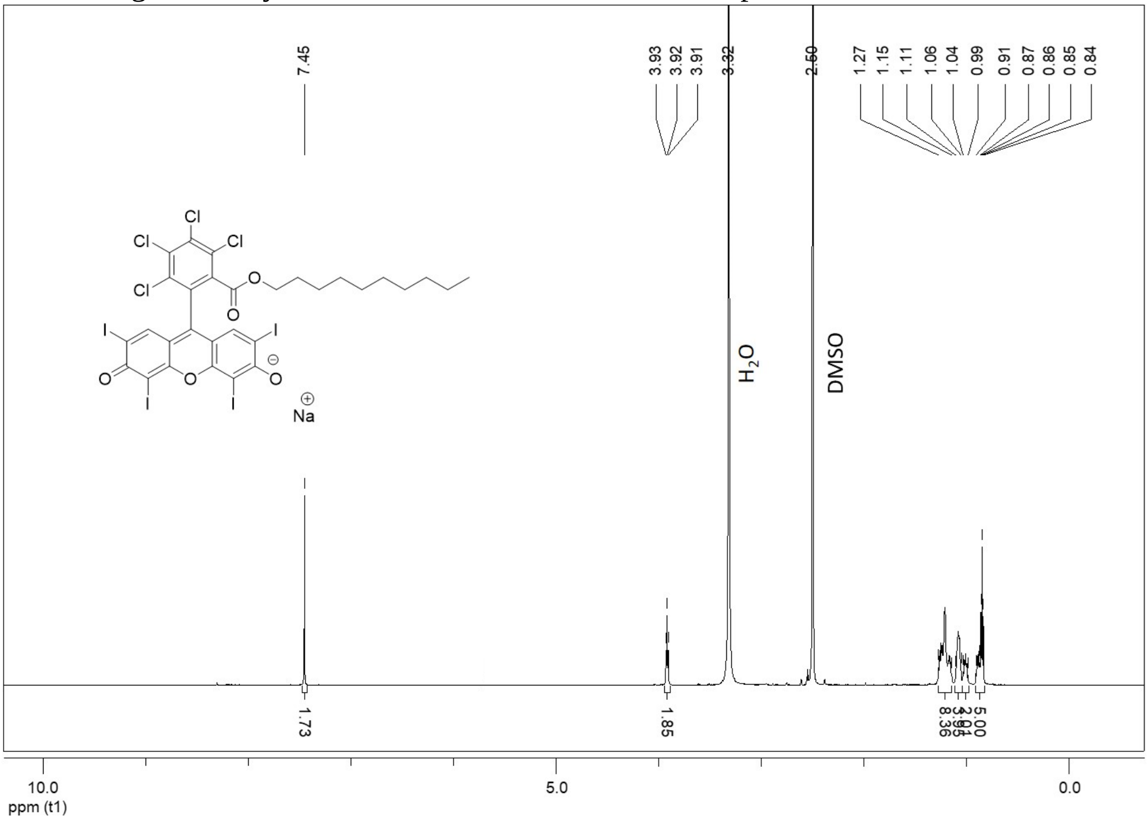


Rose Bengal n-Decyl Ester, Monosodium Salt **2d,** $^{13}$C spectrum

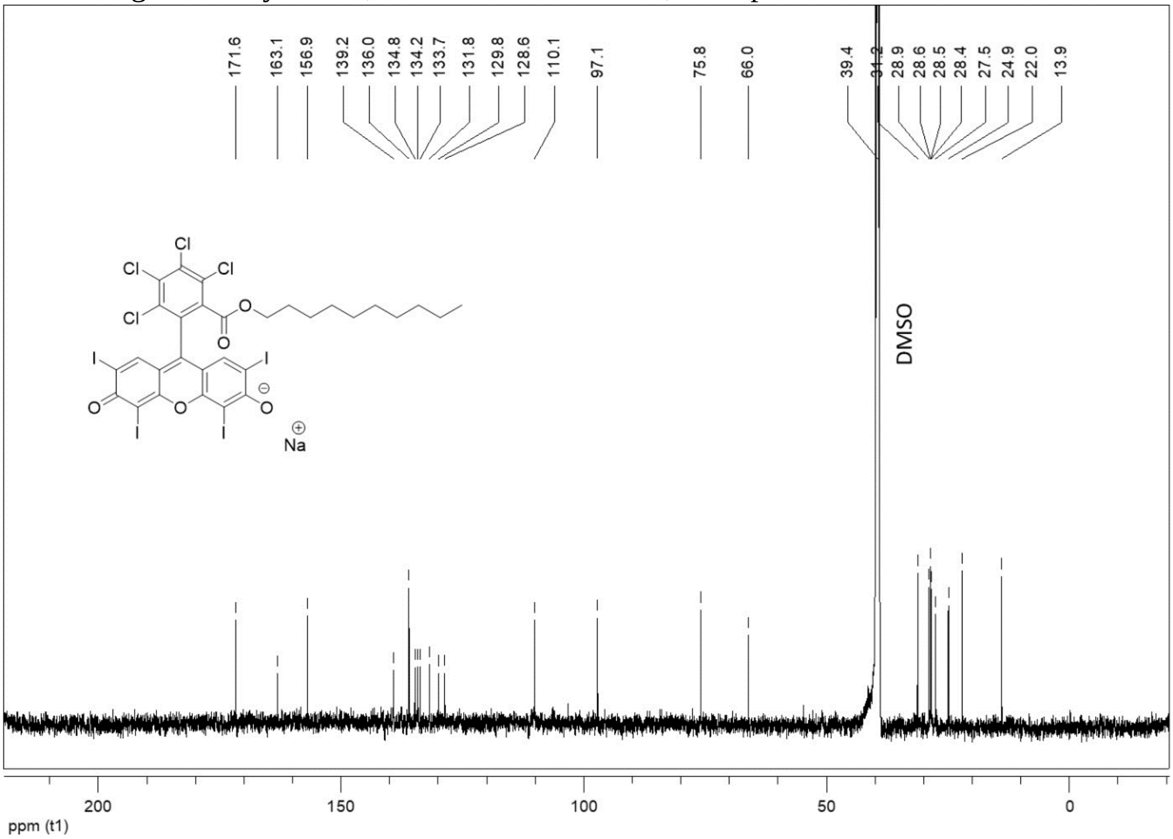


Back to Contents

Rose Bengal n-Dodecyl Ester, Monosodium Salt **2e,** $^{1}$H spectrum

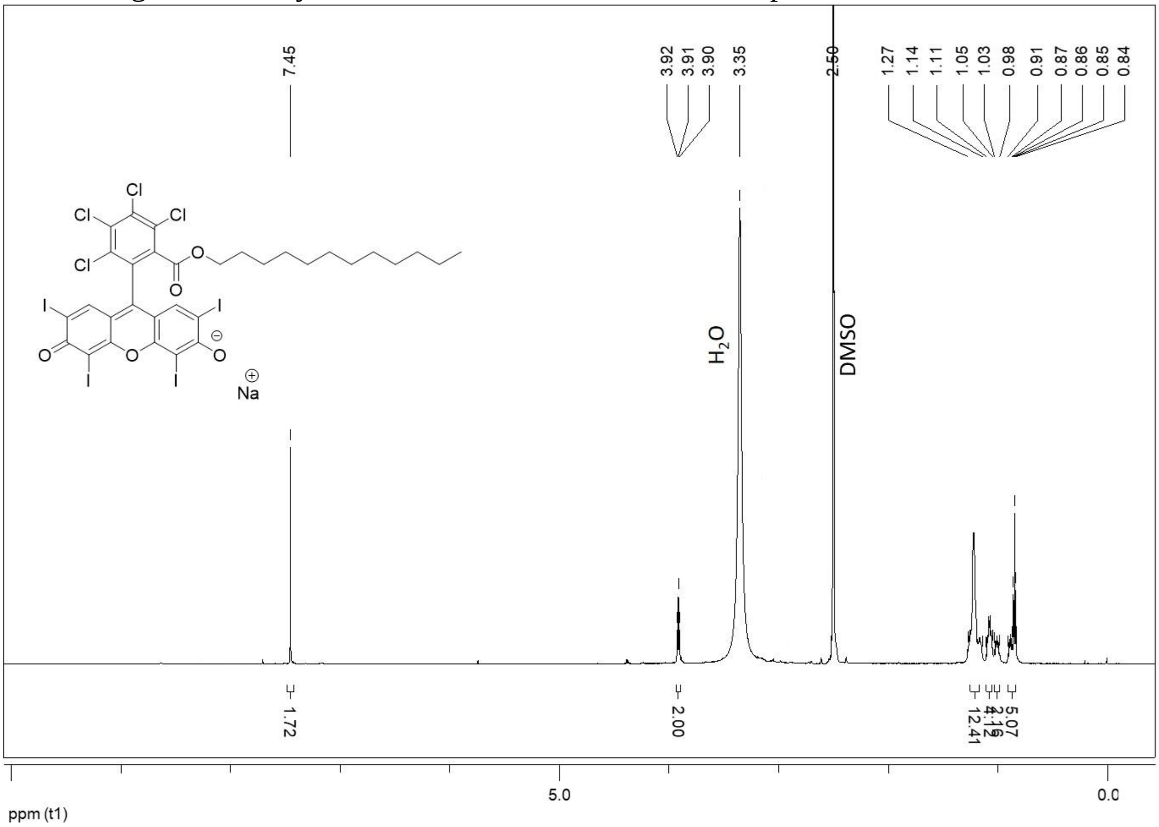


Rose Bengal n-Dodecyl Ester, Monosodium Salt **2e,** $^{13}$C spectrum

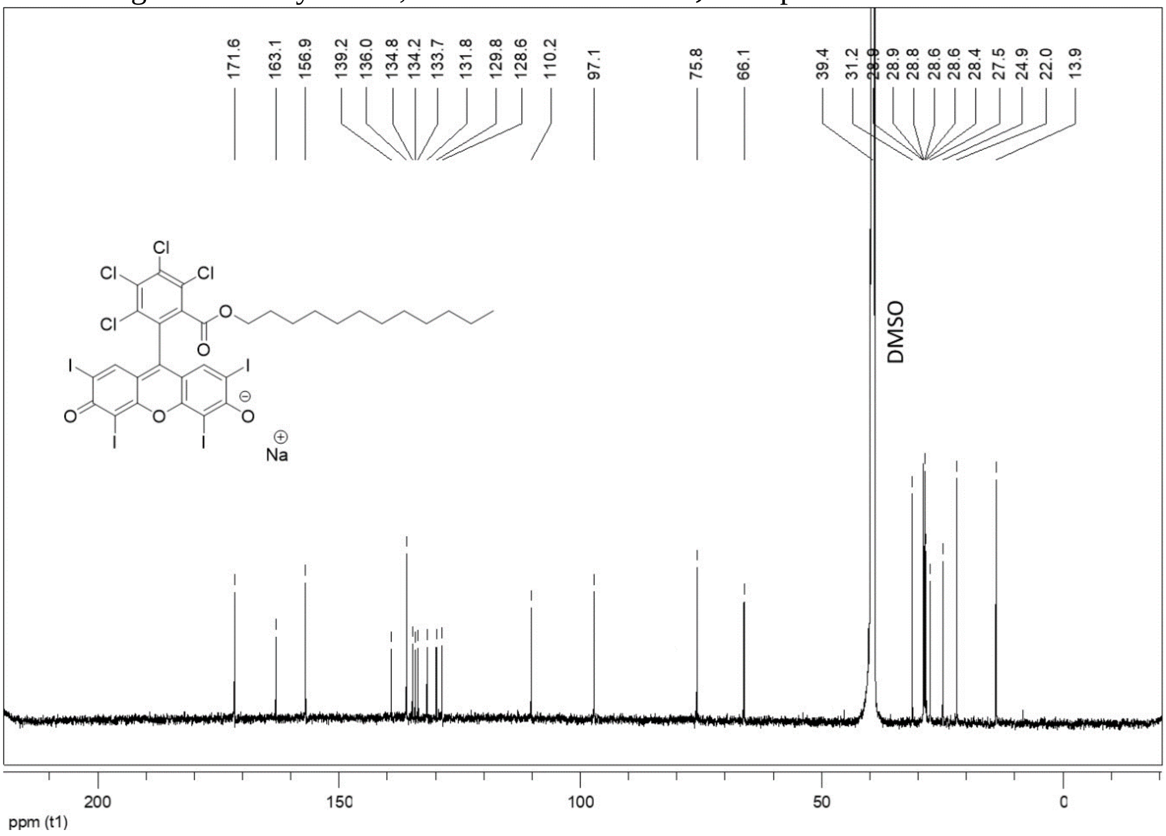


Back to Contents
